# Randomized control trial of ultrasound-guided erector spinae block versus shoulder periarticular anesthetic infiltration for pain control after arthroscopic shoulder surgery: Study protocol clinical trial (SPIRIT compliant)

**DOI:** 10.1097/MD.0000000000019721

**Published:** 2020-04-10

**Authors:** Mark Czuczman, Harsha Shanthanna, Bashar Alolabi, Peter Moisiuk, Turlough O’Hare, Moin Khan, Mauricio Forero, Kimberly Davis, Jaydeep Moro, Thuva Vanniyasingam, Lehana Thabane

**Affiliations:** a Department of Anesthesia; b Department of Anesthesia, St Joseph's Health Care; c Department of Surgery, Joseph's Healthcare; d Department of Health Research Methods, Evidence, and Impact, McMaster University, Hamilton, Ontario, Canada.

**Keywords:** arthroscopic shoulder surgery, erector spinae plane block, periarticular infiltration, postoperative pain

## Abstract

**Introduction:**

Moderate to severe postoperative pain and associated opioid use may interfere with patients’ well-being and course of recovery. Regional anesthetic techniques provide an opportunity for opioid sparing and improved patient outcomes. A new regional technique called the erector spinae plane (ESP) block has the potential to provide effective analgesia after shoulder arthroscopy with minimal risks and decreased opioid consumption. Our primary objective is to determine whether, in patients who undergo arthroscopic shoulder surgery, a preoperative ESP block reduces pain scores as compared to periarticular infiltration at the end of surgery. Additionally, we will also examine other factors such as opioid consumption, sensory block, adverse events, patient satisfaction, and persistent pain.

**Methods:**

This is a 2-arm, single-center, parallel-design, double-blind randomized controlled trial of 60 patients undergoing arthroscopic shoulder surgery. Eligible patients will be recruited in the preoperative clinic. Using a computer-generated randomization, with a 1:1 allocation ratio, patients will be randomized to either the ESP or periarticular infiltration group. Patients will be followed in hospital in the postanesthesia care unit, at 24 hours, and at 1 month. The study with be analyzed as intention-to-treat.

**Discussion:**

This study will inform an evidence-based choice in recommending ESP block for shoulder arthroscopy, as well as providing safety data. The merits of the study include its double dummy blinding to minimize observer bias, and its assessment of patient important outcomes, including pain scores, opioid consumption, and patient satisfaction. This study will also help provide an estimate of the incidence of side effects and complications of the ESP block.

**Trial registration number:**

NCT03691922; Recruited Date of registration: October 2, 2018.

## Abstract 2

Supplemental Digital Content is available in the text

## 1 Introduction

Arthroscopic shoulder surgical repairs are common procedures performed to address different shoulder pathologies. Despite the fact that it is minimally invasive, it is often associated with moderate to severe postoperative pain that may interfere with the patients’ well-being, recovery and rehabilitation, and potentially increases hospital length of stay.^[1]^ The use of opioids to manage immediate postoperative pain is frequently associated with nausea, vomiting, respiratory depression, hormonal effects, and dysphoria.^[2]^ As such, achieving pain control while minimizing opioid use is critical, since more than 60% of unplanned prolonged hospitalizations and hospital readmissions are thought to be related to inadequate pain control or to side effects of opioids.^[3]^

A number of techniques have been used to achieve good pain control after shoulder surgery, including periarticular infiltration (PAI) with local anesthetic (LA) and regional anesthetic blocks. Although shoulder PAI has been shown to decrease shoulder pain and opioid consumption,^4^,^5^,^6^ it is not as effective as regional blocks, such as the interscalene nerve block (ISNB).^7^,^8^ ISNB is considered the gold standard of regional nerve blocks for shoulder surgery but has the potential for significant side effects, such as persistent neurologic complications, rebound pain, phrenic nerve palsy, respiratory distress, cardiac arrest, pneumothorax, and central nerve toxicity.^9^,^10^,^11^,^12^,^13^ In view of this, investigating alternate regional blocks having the potential for good pain relief with minimal side effects is important.

The erector spinae plane (ESP) block can be considered as a modification of the thoracic paravertebral block, which blocks thoracic spinal nerves using injections outside of the conventional paravertebral space.^[14]^ It was first described by Forero et al in 2016 in case reports of 2 patients of severe thoracic neuropathic pain as well as 2 cases of acute postsurgical pain following video-assisted thoracoscopic wedge resection and lobectomy.^[15]^ The ESP block demonstrated a unilateral cutaneous sensory block of the posterior, lateral, and anterior chest wall as well as relief of neuropathic pain in these cases.^[15]^ ESP block is performed under ultrasound (US) by injecting LA deep to the erector spinae muscle at the interfascial space between either the erector spinae muscle and the rhomboid major muscle (higher up), or between the erector spinae muscle and the external intercostal muscles, at lower sites.^[15]^ Cadaveric studies of US-guided ESP blocks with methylene blue dye and subsequent dissection, as well an ESP block with a dye mixture and computed tomography scanning demonstrated that when injecting deep to the erector spinae the block likely affects the ventral and dorsal rami leading to the sensory blockade.^[15]^ The advantages include its simplicity and safety by limiting the risk of nerve damage and pneumothorax.^15^,^16^ Subsequent case reports have demonstrated ESP to be effective for abdominal surgery,^17^,^18^ breast and axillary surgery,^[19]^ and open radical cystoprostatectomy with ureter and bladder reconstruction.^[20]^ Recent case reports have explored its potential use for shoulder surgery when performed at a higher level (such as T2-T3). Forero et al reported successful management of chronic shoulder pain without motor blockade, with ESP performed at T3 level.^[21]^ A case report demonstrated ESP block used for shoulder surgery in 3 patients with variable success for postoperative analgesia.^[22]^

Our literature search of PubMed did not identify any randomized controlled trial (RCT) comparing ESP block to PAI or ISNB. We also looked to identify studies that have utilized ESP for post-surgical shoulder pain. There was only 1 case report of ESP for shoulder surgery.^[22]^ A search of clinicaltrial.gov revealed only 1 RCT examining ESP against ISNB for arthroscopic shoulder surgery, which is in the recruitment phase (https://clinicaltrials.gov/ct2/show/NCT03807505). Given the potential to provide effective sensory blockade with minimal technical risks, we think it is important to study the utility of ESP for arthroscopic shoulder surgeries. Currently, the ISNB is the preferred method for shoulder surgery analgesia, but it has associated risks of diaphragm paralysis and rebound pain.^23^,^24^ Given the importance of providing adequate analgesia for arthroscopic shoulder surgery and lack of consensus among surgeons and anesthesiologists for the optimal analgesic technique, we designed this RCT to compare the ESP blockade versus PAI of LA in patients undergoing arthroscopic shoulder surgery. Although it is important to establish the efficacy of ESP for shoulder surgery by comparing it with an inactive or placebo treatment, placebo injections can be considered unethical and may not be appealing to patients. On the other hand, comparing against an inactive treatment (such as no injection) can introduce potential bias and affects the internal validity of the study. Although PAI in the shoulder has been shown to decrease pain and opioid consumption,^[5]^ it is not as effective as ISNB^[7]^ and it may not be appropriate to compare it against the gold standard of ISNB. Hence, we decided to compare ESP versus PAI, which is an active treatment but not the present standard.

### 1.1 Clinical hypothesis

In patients who undergo arthroscopic shoulder surgery, “ESP block with LA” will provide superior analgesia compared to “PAI with LA.”

### 1.2 Objectives

The primary objective of this study is to determine whether ESP block is superior to PAI for in-hospital postoperative analgesia among patients who have undergone arthroscopic shoulder surgical repair. This will be determined by comparing the resting pain scores after initial stabilization in the postanesthetic care unit (PACU).

Secondary objectives will be to compare:

(1) resting pain scores at discharge;
(2) pain scores with movement in PACU and at discharge;
(3) total opioid consumption in hospital;
(4) incidences of postoperative nausea and vomiting (PONV), itching, ipsilateral diaphragmatic paralysis, respiratory depression, and LA toxicity in hospital;
(5) sensory blockade assessment in PACU;
(6) patient satisfaction at discharge,
(7) average pain scores, persistent surgical site pain and opioid use at 24 hours and at 1 month postoperatively.

## 2 Methods

### 2.1 Design

**Figure 1:**
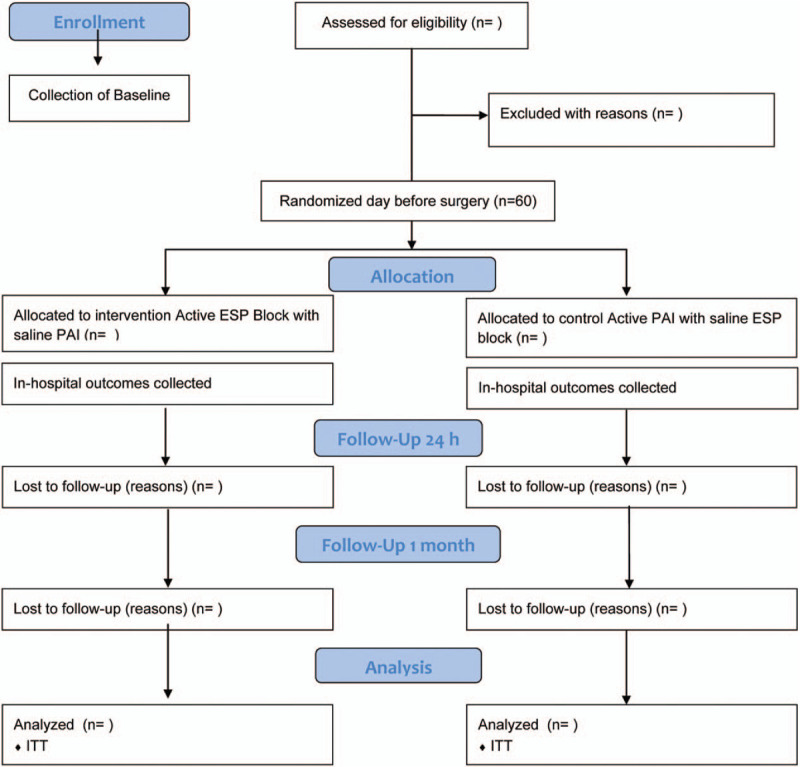
ESP versus PAI for arthroscopic shoulder surgeries study consolidated standards for reporting trials flow diagram. ESP = erector spinae plane block, PAI = periarticular infiltration.

This will be a single-center RCT with a 2-arm parallel design (Fig. 1) and will be conducted at the St. Joseph's Healthcare Hamilton, affiliated with McMaster University, Canada.

### 2.2 Patient selection

Eligible patients will be screened during their preanesthetic visit before their surgical date by a trained research assistant using the following selection criteria. Anesthesiologists at the clinic will offer postoperative pain management strategies including the study interventions. Informed consent of willing participants will be obtained along with their baseline parameters. Patients will have the opportunity to withdraw from the study at any point during the surgery or the postoperative phase until their discharge. Baseline parameters will include age, sex, height, weight, body mass index, and the American Society of Anesthesiologists classification.

#### 2.2.1 Inclusion criteria

Patients aged >18 years undergoing elective arthroscopic shoulder joint repairs admitted for a day surgical procedure, with the ability to provide informed consent.

#### 2.2.2 Exclusion criteria

Not consenting; contraindications to spinal injections as per the American Society of Regional Anesthesia and Pain guidelines^[25]^; known allergy to LA; allergy to all opioid medications; diagnostic shoulder arthroscopic procedures; inability to understand or comprehend in English language; history of daily opioid medication use for the past 1 month; and patients with planned overnight hospital stay.

### 2.3 Control of bias

#### 2.3.1 Randomization

Treatment allocation will be done with an allocation ratio of 1:1 using a computer-generated variable block randomization using block sizes of 2, 4, and 6. This randomization list will be prepared by a statistician who is not involved with the study data analysis and provided to the hospital pharmacy. Randomization will happen on the day before surgery. Each patient will be designated a unique randomization code.

#### 2.3.2 Allocation concealment

Study allocation will be performed by the pharmacy and will be concealed by having 2 sets of syringes with equal volumes marked as “for ESPB” and “for PAI” delivered to the operating room (OR) on the day of surgery, before the performance of the block for each patient. The solutions within the syringes will not have any identifiers, and hence will be completely concealed. These syringes will be used for the respective study interventions for each patient.

#### 2.3.3 Achievement of blinding

Since the medication syringes contain clear solutions, and the study uses a double dummy technique for blinding by performing both interventions in each patient, patients, physicians, other healthcare providers, and data collectors are effectively blinded. Apart from the statistician who has prepared the randomization code and the pharmacist preparing the study medications, all other personnel are blinded for assessment and data analysis.

### 2.4 Application of interventions

#### 2.4.1 Intervention group

##### 2.4.1.1 Active ESP block, saline PAI

The ESP block will be performed immediately before surgery, in a designated regional anesthesia block room. Intravenous (IV) access will be established and standard monitoring including noninvasive blood pressure, electrocardiogram, and pulse oximetry will be applied. Sedation and anxiolytics may be used as clinically appropriate. The block will be performed by anesthesiologists who have been trained to perform ESP and who do ESP on a regular basis. The patient will be placed in a sitting position and the area over the thoracic spine will be sterilized with disposable swabs of 2% chlorhexidine in 70% isopropyl alcohol and then draped in a sterile fashion. A high-frequency linear US transducer (GE. LOGIQe) will be placed in a longitudinal parasagittal orientation 3 cm lateral to the T2 spinous process. The trapezius, rhomboid major, and erector spinae muscles will then be identified superficial to the tip of the T2 transverse process. The patient's skin will be anesthetized with 2% lidocaine. A 17-gauge 8 cm needle (Arrow StimuCath; Teleflex Medical, Markham, Ontario, Canada) will be inserted using an in-plane superior-to-inferior approach to place the tip into the fascial plane on the deep (anterior) aspect of erector spinae muscle. The location of the needle tip will be confirmed by visible fluid spread lifting erector spinae muscle off the bony shadow of the transverse process. A total of 30 mL of 0.25% bupivacaine with 5 μg/mL of epinephrine will be injected in 5 mL aliquots through the needle (maximum of 3 mg/kg).

The orthopedic surgeon will perform saline PAI at the end of surgical procedure on the operated side rotator cuff. The surgeons will identify the desired area and advance the needle at an approximately 20° angle in the joint space. The PAI will be performed using a total of 30 mL of saline injected in 5 mL aliquots through the needle. Nurse-administered opioids will be initiated postoperatively in the PACU.

#### 2.4.2 Control group

##### 2.4.2.1 Saline ESP block, active PAI

The ESP block will be performed similar to above, but with 30 mL of saline injected as 5 mL aliquots, immediately before surgery. The orthopedic surgeon will perform an active PAI at the end of surgical procedure, on the operated side rotator cuff using 30 mL of 0.25% bupivacaine with 5 μg/mL of epinephrine injected in 5 mL aliquots. The procedure will be performed as described above.

#### 2.4.3 The OR

Patients included in the study would be managed according to OR protocol. Study patients will be identified during the surgical “time out” period. The attending anesthesiologist responsible for the care of the patient will provide a general anesthetic as per the routine institutional practice. Both groups will have no restrictions on the anesthetic management except that any long-acting agent (morphine/hydromorphone) is administered at least 30 minutes before extubation, so that the PACU pain scores are comparable.

#### 2.4.4 PACU and day surgery unit (DSU) protocol

All patients will be monitored in the PACU until they are transferred to DSU before their discharge. Standard order sets will be used for PACU and DSU analgesia. A research assistant will be present in PACU or DSU for all patients at all times. For both groups, nurse-administered IV opioids will be initiated in PACU similar to routine practice according to the order sets (choice of fentanyl, hydromorphone, or morphine). Pain scores will be appropriately recorded and pain scores at 30 minutes after PACU admission will be noted for analysis. Opioid analgesic administration will be individualized based on allergies, co-morbidities, and patient tolerance. Ketorolac will be used only as a rescue (30 mg IV) for pain score of >3/10 after 30 minutes (as appropriate based on allergy). At 30 minutes in PACU, patients will receive acetaminophen if they can tolerate by mouth (as appropriate based on allergy). Patients will be discharged home when DSU discharge criteria are met. Standardized prescriptions will be given to each patient based on individual allergies and comorbidities.

#### 2.4.5 Follow-up

Study patients will be followed for 24 hours and at 1 month after surgery by a telephone call from a trained research assistant with a scripted set of questions. Participant flow is shown in Figure 1 (consolidated standards of reporting trials flow chart).

### 2.5 Outcomes

#### 2.5.1 Primary outcome and measurement

The primary outcome of this study will be to compare the resting pain scores after initial stabilization (30 minutes after admission) in PACU. Pain scores will be noted using the patient-reported numeric rating scale (NRS), an 11-point scale where 0 is no pain and 10 the worst pain imaginable.^[26]^ NRS is validated and considered easy to use.^[27]^

#### 2.5.2 Secondary outcomes and measurement

The following secondary outcomes will be collected during the in-hospital stay and follow-up of study patients:

(1) Total opioid consumption in hospital (until discharge): opioid use will be converted into morphine equivalent dosage for analysis.
(2) Pain intensity: pain scores with movement in PACU; pain scores at rest; and movement at readiness to discharge. Since patients will be asked not to move their shoulders by the surgical team, pain scores with movement will be collected by asking the patient to sit up from lying down position. Pain scores will be collected using the 11-point NRS scale.^[26]^ We will record the postoperative time of peak pain scores.
(3) Safety outcomes: incidence of moderate to severe PONV and incidence of moderate to severe itching will be collected using validated scales^28^,^29^; incidence of ipsilateral diaphragmatic paralysis will be diagnosed using bedside US examination with sniff test,^[30]^ 30 minutes after admission. This has been observed to be valid and clinically feasible^[31]^; and incidence of respiratory depression^[32]^ and LA toxicity^[33]^ will be diagnosed based on clinical symptoms and signs. All these outcomes will be noted postoperatively in PACU and until discharge, unless specified.
(4) Sensory blockade on the side of the operated shoulder: Sensory assessment of shoulder area will be performed by a trained research assistant. Sensory dermatomes corresponding to shoulder and upper arm and their blockade to cold sensation will be noted. In total we will observe sensory blockade in 7 dermatomal areas (Supplemental Digital Content (Appendix 1)) and compare the extent of blockade between the 2 groups by their median and range.^[34]^
(5) Patient satisfaction with postoperative analgesia: This will be recorded at hospital discharge and 24 hours and at 1 month after surgery (by a telephone call) and measured using a 7-item Likert scale.

#### 2.5.3 Outcomes at 24 hours

Patients will be contacted by telephone the next day, 24 hours after surgery, to ask the following questions:

(1) What was your average pain score over the last 24 hours after discharge, on a scale of 0 to 10 (NRS)?
(2) Rebound pain: Is the nerve block still providing pain relief? Yes or No
  At what time following the surgery did the block wear off?
  At what time following the surgery did you take your first dose of opioid?
(3) Are you continuing to use opioids postoperatively? If yes, provide drug name, frequency, and amount over the past 24 hours.
(4) Are you satisfied with your postoperative pain control (Likert scale 1–7)?

#### 2.5.4 Outcomes at 1 month

Patients will be contacted by telephone at 1 month to ask the following questions:

(1) Are you experiencing any persisting pain at the site of surgery? If yes, please provide details.
(2) What is your average daily pain score, on a scale of 0 to 10 (NRS)?
(3) Are you continuing to use opioids postoperatively? If yes, provide drug name, frequency and amount over the past 24 hours.
(4) How many days per week opioids are used at least once for operative pain?
(5) Are you satisfied with your postoperative pain control (Likert scale 1–7)?

### 2.6 Analysis

#### 2.6.1 Sample size estimation (Table 1)

Existing literature suggests that single-shot ISNBs with general anesthetic could achieve mean postoperative recovery pain scores of 2 to 3 (on a 0–10 visual analog scale),^[35]^ and patients having general anesthetic with PAI block for shoulder surgery report pain scores of 5 to 6 (on a 0–10 NRS) at PACU.^5^,^36^,^37^ We considered a reduction in mean pain scores by 2 points to be clinically meaningful and important. We believe the ESP block has the potential to provide a good quality of analgesia, nearly similar to ISNB with less chance of phrenic nerve blockade. Based on these assumptions, we considered the following for our sample size analysis. For a 2-sided test, assuming a mean pain score of 5.5 (standard deviation of 2.5) in the control (PAI) group, a sample size of 25 per group will have 80% power to detect a statistically significant difference in mean pain scores of 2 or more using a Student *t* test, with an alpha set at 0.05. We expect minimal loss through attrition as the observations happen in hospital before discharge. However, considering the possibility of changes to surgical plan, we decided to set the sample size at 30 per group (which could give us 85% power). Sample size was estimated using (http://biostat.mc.vanderbilt.edu/wiki/Main/PowerSampleSize#PS:_Power_and_Sample_Size_Calculation), version 3.0.43.

**Table 1:**
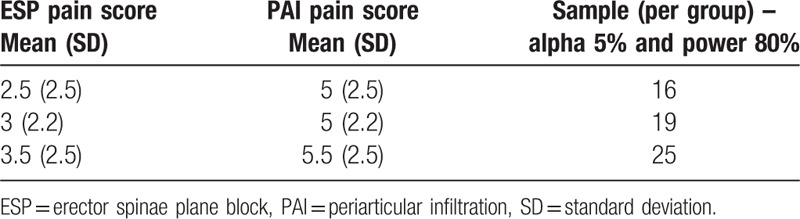
Sample size estimation table.

#### 2.6.2 Statistical analysis

The trial will be reported as per the consolidated standards of reporting trials standards for reporting randomized trials.^[38]^ The study will be analyzed using an intention-to-treat (ITT) approach. For ITT, we will analyze patients within their randomized groups. We will use multiple imputation strategy to account for missing outcomes in ITT. Normally distributed continuous data will be reported as means and standard deviations; skewed continuous data will be reported as medians and quartiles (Q1 and Q3); and nominal data, categorical or binary data will be reported as counts and percentages. Comparison of normal continuous outcomes will be performed using Student *t* test for unpaired groups. Comparison of non-normal or skewed continuous outcomes will be performed using Mann–Whitney *U* test. Categorical outcomes will be compared using Pearson Chi-squared test. Each test will be 2-sided with a significance level of 0.05. Up-to-date versions of SAS (Cary, NC) and SPSS (Chicago, IL) will be used to conduct all analyses. Dichotomous outcomes will be reported as relative risk and relative risk reductions and continuous outcomes as difference in means with standard deviations. Precision will be reported using 95% confidence intervals. No subgroup tests will be conducted. Individual outcomes and their analyses are summarized in Table 2.

**Table 2:**
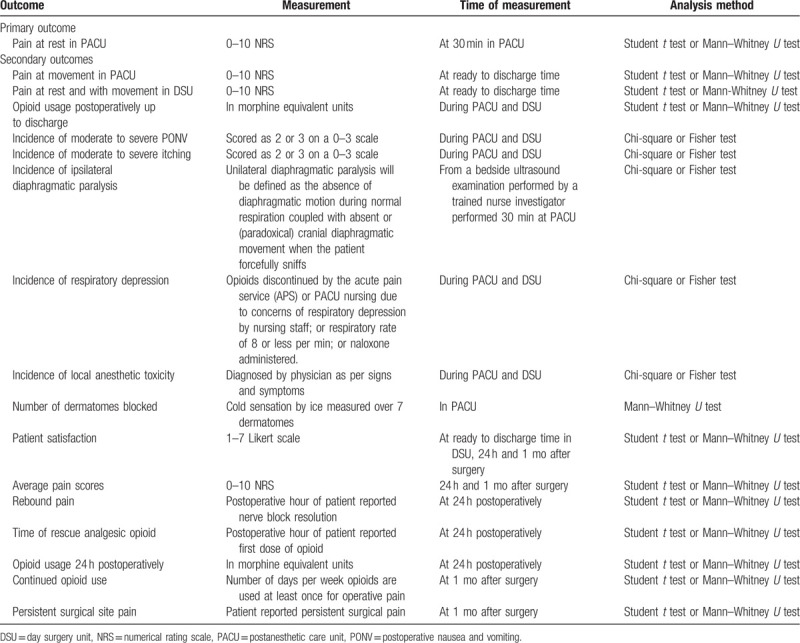
List of outcomes, measurement, and analysis.

### 2.7 Project coordination and reporting

This trial will be coordinated from the research office at the McMaster Department of Anesthesia and conducted at St. Joseph's Healthcare Hamilton.

### 2.8 Data management and quality control

Data collection will be done using paper forms and transferred to REDCap for secure storage and analysis. REDCap is a secure web application developed to optimize data collection and management for research studies. All patients will receive a study ID to keep their information anonymous. Data will be collected by research assistants and nurses in the PACU and DSU and after discharge. The research coordinator will monitor the REDCap and ensure that the data are appropriately entered, verified, and kept secure. Only the research assistants involved with the study, the coordinator, and the study statistician will have access to the patient data. The REDCap database will be stored on a server in a data center located within the McMaster University campus. The data collection forms will also be kept in the locked cabinet.

### 2.9 Risk assessment and protocol adherence

This trial does not entail any higher risk than the standard of care to patients. Medications of known benefit are used in clinically acceptable doses. Patients are managed using standard preoperative, OR, PACU, and DSU protocols. The ESP block will be performed by anesthesiologists competent with the block.

### 2.10 Patient and public involvement

Patients and public were not directly involved in the development of the study protocol. We will disseminate the results to the study participants through the journal publication as well as from our research website.

## 3 Discussion

This RCT examines ESP block versus PAI in patients undergoing shoulder arthroscopy and assesses differences in pain scores and opioid consumption, as well as in safety and other patient outcomes. It will allow physicians to make an evidence-based choice in recommending ESP block for shoulder arthroscopy, and will provide data regarding the safety profile of ESP block. The advantages of the study would be establishing efficacy of the ESP block for shoulder arthroscopy, which could include lower pain scores, less opioid consumption and better patient satisfaction in PACU, at discharge and at 1 month. This study will also help provide an estimate of the incidence of side effects and complications of the ESP block including PONV, pruritis, diaphragmatic paralysis, and LA systemic toxicity.

### 3.1 Potential pitfalls

The primary outcome is the subjective pain scores using NRS scales. These are validated scales, although they do possess some inherent limitations. For equianalgesic dose ratio and conversion to morphine equivalent units we have used the Faculty of Pain Medicine of the Australian and New Zealand College of Anaesthetists document, although other ratios and scales have been reported in the literature (http://fpm.anzca.edu.au/documents/opioid-dose-equivalence.pdf).

### 3.2 Ethics and dissemination

The study has been approved by the Hamilton Integrated Research Ethics Board. We plan to report and publish our study findings in a high-impact medical journal, with online access. We also plan to present it at selected conferences and scientific meetings.

## Acknowledgments

The authors thank Toni Tidy for their contributions as research coordinators working for this project. We would also like to thank Christian Sirko for his contributions with database setup and data collection. They sincerely thank their research pharmacist Christine Wallace for her assistance. They also thank the dedicated team of nurses working in the PACU and the DSU for their involvement and support in this study.

## Author contributions

**Conceptualization:** Mark Czuczman, Harsha Shanthanna, Bashar Alolabi, Peter Moisiuk, Turlough O’Hare, Moin Khan, Mauricio Forero, Jaydeep Moro.

**Formal analysis:** Thuva Vanniyasingam.

**Funding acquisition:** Mark Czuczman, Harsha Shanthanna, Bashar Alolabi.

**Investigation:** Mark Czuczman, Peter Moisiuk, Turlough O’Hare, Moin Khan, Mauricio Forero, Kimberly Davis, Jaydeep Moro.

**Methodology:** Lehana Thabane.

**Supervision:** Bashar Alolabi, Harsha Shanthanna.

**Writing – original draft:** Mark Czuczman, Harsha Shanthanna, Bashar Alolabi.

**Writing – review & editing:** Mark Czuczman, Harsha Shanthanna, Bashar Alolabi, Peter Moisiuk, Turlough O’Hare, Moin Khan, Mauricio Forero, Kimberly Davis, Jaydeep Moro, Thuva Vanniyasingam, Lehana Thabane.

Harsha Shanthanna orcid: 0000-0002-4105-4465.

## Supplementary Material

Supplemental Digital Content
