# Supplementary material for: Randomized control trial of ultrasound-guided erector spinae block versus shoulder periarticular anesthetic infiltration for pain control after arthroscopic shoulder surgery: Study protocol clinical trial (SPIRIT compliant)
Source: Medicine (Baltimore). 2020 Apr 10;99(15):e19721. doi: 10.1097/MD.0000000000019721 (PMC7220186; doi:10.1097/MD.0000000000019721)
Supplement: Supplemental Digital Content [file medi-99-e19721-s001.doc]

Appendix 1
